# Reactivation of the homeotic tumor suppressor gene CDX2 by 5-aza-2′-deoxycytidine-induced demethylation inhibits cell proliferation and induces caspase-independent apoptosis in gastric cancer cells

**DOI:** 10.3892/etm.2013.901

**Published:** 2013-01-17

**Authors:** JIAN-FENG ZHANG, JIAN-GUO ZHANG, XIAO-LING KUAI, HONG ZHANG, WEI JIANG, WEI-FENG DING, ZENG-LI LI, HUI-JUN ZHU, ZHEN-BIAO MAO

**Affiliations:** 1 Department of Gastroenterology, Affiliated Hospital of Nantong University, Nantong, Jiangsu 226001, P.R. China; 2 Department of Pathology; Affiliated Hospital of Nantong University, Nantong, Jiangsu 226001, P.R. China; 3 Department of Medical Laboratory Center; Affiliated Hospital of Nantong University, Nantong, Jiangsu 226001, P.R. China; 4 Department of Nephrology, Affiliated Hospital of Nantong University, Nantong, Jiangsu 226001, P.R. China

**Keywords:** gastric neoplasms, caudal type homeobox transcription factor 2, DNA methylation, DNA methyltransferase enzyme 1, proliferation, apoptosis

## Abstract

The DNA methylation inhibitor 5-aza-2′-deoxycytidine (5-aza-CdR) is widely used as an anticancer drug for the treatment of leukemia and solid tumors. Gastric cancer (GC) patients who were positive for caudal type homeobox transcription factor 2 (CDX2) expression showed a higher survival rate compared with those who were CDX2 negative, which suggests that CDX2 performs a tumor suppressor role. However, the molecular mechanisms leading to the inactivation of CDX2 remain unclear. In the present study we demonstrated that the expression levels of CDX2 and DNA methyltransferase enzyme 1 (DNMT1) mRNA were significantly higher in GC compared with distal non-cancerous tissue. The expression of CDX2 mRNA was significantly correlated with Lauren classification, TNM stage and lymph node metastasis. DNMT1 mRNA expression was significantly correlated with TNM stage, pathological differentiation and lymph node metastasis. The expression of CDX2 mRNA was inversely correlated with that of DNMT1 mRNA in GC. Hypermethylation of the CDX2 gene promoter region, extremely low expression levels of CDX2 mRNA and no expression of CDX2 protein were the characteristics observed in MKN-45 and SGC-7901 GC cell lines. Following the treatment of MKN-45 cells with 5-aza-CdR, the hypermethylated CDX2 gene promoter region was demethylated and expression of CDX2 was upregulated, while DNMT1 expression was downregulated. Furthermore, a concentration- and time-dependent growth inhibition as well as increased apoptosis were observed. Caspase-3, −8 and −9 activities increased in a concentration-dependent manner following exposure to different concentrations of 5-aza-CdR. Therefore, our data show that the overexpression of DNMT1 and methylation of the CDX2 gene promoter region is likely to be responsible for CDX2 silencing in GC. 5-Aza-CdR may effectively induce re-expression of the CDX2 gene, inhibit cell proliferation and enhance the caspase-independent apoptosis of MKN-45 cells *in vitro*.

## Introduction

Gastric cancer (GC) is a worldwide health problem with >600,000 cases reported annually. The highest rates occur in Japan, China, Eastern Europe and South America, with 42% of worldwide cases occurring in China (1,2). The occurrence of GC is associated with gene mutations, deletions and other genetic and epigenetic mechanisms, which are the result of the interaction between genetic and environmental factors. Epigenetic modification controls gene expression via DNA methylation, histone modification, chromatin remodeling and non-coding RNAs. Abnormal epigenetic modifications may lead to tumorigenesis (3,4), and their role in the process of tumor formation is likely to be of increasing interest. DNA methylation is a type of reaction using S-adenosylmethionine (SAM) as a methyl donor for converting cytosine into S-methyl cytosine using the DNA methyltransferase enzyme (DNMT) (5). Mizuno *et al*(6) showed that the expression of DNA methyltransferase 1 (DNMT1) in tumor cells is 4-12-fold higher than that in normal cells, confirming that the DNMT1 increase is involved in tumorigenesis. 5-Aza-2′-deoxycytidine (5-aza-CdR) is a nucleoside analog methylation inhibitor that forms a covalent complex with DNMT1 to inhibit its methyltransferase activity, resulting in less methylation (7).

Caudal type homeobox transcription factor 2 (CDX2), a member of the caudal-related homeobox gene family, plays a key role in early mammalian intestinal development and the maintenance of intestinal epithelia via regulation of intestine-specific gene transcription (8,9). CDX2 expression in normal tissue mainly exists in the small intestinal and colonic mucosa, not in the normal gastric mucosa, but it appears ectopic expression from intestinal metaplasia to the intestinal-type GC (10). Numerous studies indicate that CDX2 performs a tumor suppressor role in human colorectal carcinogenesis (11–14). Recently, several studies reported that the survival rates for CDX2 expression-positive GC were significantly higher than those for the negative group, suggesting a possible tumor suppressor role for CDX2 (15,16). However, the molecular mechanisms leading to the inactivation of CDX2 remain unclear.

In the present study, the expression and association of CDX2 and DNMT1 mRNA in GC tissues and normal tissues distal to the GC were analyzed. To further elucidate the molecular mechanisms behind the inactivation of the CDX2 gene, we detected the promoter methylation status by methylation-specific PCR (MSP) and the expression of CDX2 mRNA and protein by real-time fluorescence quantitative polymerase chain reaction (RFQ-PCR) and western blotting in the GC cell lines AGS, MKN-45 and SGC-7901. We then investigated the ability of 5-aza-CdR to induce CDX2 gene re-expression and its effects and mechanisms in GC cell proliferation and apoptosis by treating MKN-45 cells with the demethylating agent 5-aza-CdR *in vitro*. Our study reveals that the loss of CDX2 function in GC may be attributed to promoter hypermethylation, and that the reactivation of CDX2 by 5-aza-CdR inhibits cell proliferation and induces caspase-3-independent apoptosis in GC cells.

## Materials and methods

### Tissue samples

Sixty pairs of tissue specimens of GC and the matching distal non-cancerous gastric mucosal tissues (the distance from normal tissue to tumor was >5 cm) were obtained from 60 patients. All patients underwent surgery without preoperative radiation or chemotherapy at the Surgery Department of the Affiliated Hospital of Nantong University (Nantong, China) between January 2009 and August 2011. The tumor and the distal non-cancerous gastric mucosal tissues were snap frozen in liquid nitrogen (N_2_) and stored at −80°C until use. All the specimens were diagnosed separately by two pathologists to determine the pathological classification of GC according to the 7th edition of the AJCC cancer staging manual for stomach cancer (17). The detailed profiles of clinical and pathological variables, including age, gender, tumor size, Lauren classification, differentiation and lymph node metastasis status of the patients, are listed in Tables I and II. The tissue samples were collected with the informed consent of all patients and approval of the ethics committee of the Affiliated Hospital of Nantong University.

### Cell culture and 5-aza-CdR treatment

The human GC cell lines MKN-45, AGS and SGC-7901, purchased from the Cell Resource Center of Shanghai Institutes for Biological Sciences affiliated to the Chinese Academy of Sciences (Shanghai, China) were maintained in RPMI-1640 medium (GIBCO, Grand Island, NY, USA), supplemented with 10% heat-inactivated fetal bovine serum (Invitrogen, Carlsbad, CA, USA) and 1% antibiotic-antimycotic (Invitrogen), and grown at 37°C in a humidified atmosphere containing 5% CO_2_. The cell lines were treated with 2.5, 5 or 10 *μ*mol/l 5-aza-CdR (Sigma, St. Louis, MO, USA) for 24, 48 or 72 h.

### RFQ-PCR

Total RNA from cultured cells and tissue samples was isolated using TRIzol reagent (Takara, Dalian, Japan) according to the manufacturer’s instructions. Primer pairs designed by Premier 5.0 (Premier Biosoft, Palo Alto, CA, USA) were synthesized by Sangon Biotech (Shanghai) Co. Ltd (Shanghai, China) The primers were as follows: CDX2, forward: 5′-CGC CGC AGA ACT TCG TCA G-3′ and reverse: 5′-CGT AGC CAT TCC AGT CCT CCC-3′; DNMT1, forward: 5′-CTA CCA GGG AGA AGG ACA GG-3′ and reverse: 5′-CTC ACA GAC GCC ACA TCG-3′; β-actin (used as an internal control), forward: 5′-TGA CGT GGA CAT CCG CAA AG-3′ and reverse: 5′-CTG GAA GGT GGA CAG CGA GG-3′. Polymerase chain reaction (PCR) system: 10 *μ*l SYBR Premix Ex Taq^™^ (2X), 0.4 *μ*l PCR forward primer, 0.4 *μ*l PCR reverse primer, 2.0 *μ*l PCR template (cDNA solution), 0.4 *μ*l ROX Reference Dye (50X) and 6.8 *μ*l sterile double steamed water. The mRNA was amplified for 40 cycles and the cycling parameters were: 95°C for 30 sec, 95°C for 5 sec and 60°C for 34 sec. Each measurement was performed in triplicate and the average was calculated. For relative quantification, 2^−ΔΔCt^ was calculated and used as an indication of the relative expression levels (18).

### Western blotting

Western blotting analysis of the CDX2 and internal control β-actin proteins was performed as described previously (19). Monoclonal antibody to CDX2 protein (Santa Cruz Biotechnology, Santa Cruz, CA, USA) was diluted at 1:100.

### DNA isolation and MSP

The DNA was extracted using a DNeasy Blood & Tissue kit (Qiagen, New York, NY, USA). The DNA (2 *μ*g) was modified using an EpiTect Bisulfite kit (Qiagen). Methylation of the CDX2 gene CpG islands was analyzed by an MSP procedure, as previously described (20). The primers and PCR conditions have been described previously (21). The primers were as follows: CDX2 methylated sense: 5′-CGT CGG TTT GGG GTT TCG TAC-3′; antisense: 5′-GAT ACT CCG CTA ACT CCT CGC G-3′, expected fragment length, 169 bp; CDX2 unmethylated sense: 5′-GAA GTT GTT GGT TTG GGG TTT TGT AT-3′; antisense: 5′-CCC ACA ATA CTC CAC TAA CTC CTC ACA-3′, expected fragment length, 180 bp. The PCR products were electrophoresed in 2.5% agarose gels. The MSP procedures were performed in triplicate.

### Cell proliferation assay

Cell proliferation was determined using a WST-8 Cell Counting Kit-8 (CCK-8; Dojindo Laboratories, Kunamoto, Japan) according to the manufacturer’s instructions. Briefly, cells (5×10^7^ cells/l) suspended in RPMI-1640 medium (100 *μ*l) containing 10% fetal bovine serum were seeded in 96-well plates and incubated for 24, 48, 72 and 96 h. CCK-8 solution (10 *μ*l) was added to each well and the cultures were incubated at 37°C for 3 h. Absorbance at 450 nm was measured using an immunoreader. The results were plotted as means ± standard deviation of three separate experiments having four determinations per experiment for each experimental condition.

### Annexin V-FITC/propidium iodide (PI) assay

Cell apoptosis was analyzed by flow cytometry with an Annexin V-FITC Apoptosis Detection kit (Beyotime, Jiangshu, China) according to the manufacturer’s instructions. Briefly, MKN-45 cells were treated with 0, 2.5, 5 or 10 *μ*mol/l 5-aza-CdR for 72 h, then collected and washed twice with cold phosphate-buffered saline (PBS). Following the addition of 195 *μ*l binding buffer, 5 *μ*l FITC-labeled annexin V was added and the cells were incubated for 10 min at room temperature. Each sample was then centrifuged at 1000 x g for 5 min, resuspended in 190 *μ*l binding buffer and 10 *μ*l PI working solution was added. The samples were analyzed by flow cytometry (FCM).

### Hoechst 33258 staining

Morphological observation of nuclear change was assayed with Hoechst 33258 staining (Beyotime) according to the manufacturer’s instructions. MKN-45 cells (1×10^6^cells/ml) were seeded in 6-well plates and treated with 0, 2.5, 5 or 10 *μ*mol/l 5-aza-CdR for 72 h at 37°C. The cells were collected, washed and fixed in 4% paraformaldehyde for 30 min and then stained with 5 *μ*g/ml Hoechst 33258 for 5 min at room temperature. The apoptotic cells were visualized using an inverted fluorescence microscope (Olympus, Tokyo, Japan).

### Analysis of caspase activities

Caspase activities were measured using caspase activity assay kits C1115, C1151 and C1157 (Beyotime) according to the manufacturer’s instructions. Briefly, cells were washed with PBS, resuspended in lysis buffer and left on ice for 15 min. The lysate was centrifuged at 20,000 x g at 4°C for 15 min. The activities of caspase-3, −8 and −9 were measured using substrate peptides acetyl-Asp-Glu-Val-Asp p-nitroanilide (Ac-DEVD-pNA), acetyl-Ile-Glu-Thr-Asp p-nitroanilide (Ac-IETD-pNA) and acetyl-Leu-Glu-His-Asp p-nitroanilide (Ac-LEHD-pNA), respectively. The release of p-nitroanilide (pNA) was qualified by determining the absorbance with a microplate reader (Model 550, Bio-Rad, Hercules, CA, USA) at 405 nm. Each plate contained multiple wells of a given experimental condition and multiple control wells.

### Statistical analysis

Results were presented as the mean ± standard deviation. All data were analyzed using statistical software Stata version 11.0 (Stata, College Station, TX, USA). Statistical differences between the groups were analyzed using either one-way ANOVA or Student’s t-test. Linear regression was calculated between the expression levels of CDX2 and DNMT1 mRNA in tissues. P<0.05 was considered to indicate a statistically significant result.

## Results

### Expression of CDX2 and DNMT1 mRNA in GC and distal non-cancerous gastric tissues

The expression levels of CDX2 and DNMT1 mRNA in 60 GC tissue samples and the matching non-cancerous gastric mucosa tissue samples was detected by RFQ-PCR. The expression levels of CDX2 and DNMT1 mRNA were significantly higher in the GC tissues than in the non-cancerous tissues (P<0.05). The expression of CDX2 mRNA was significantly correlated with Lauren classification, TNM stage and lymph node metastasis (all P<0.05). DNMT1 mRNA expression was significantly correlated with TNM stage, pathological differentiation and lymph node metastasis (all P<0.05; Tables I and II). Linear correlation analysis showed that the expression of CDX2 mRNA was inversely correlated with that of DNMT1 mRNA in GC (r=−0.385, P<0.05).

### Association between methylation of the CDX2 gene promoter and gene expression in GC cells

**Figure 1.**
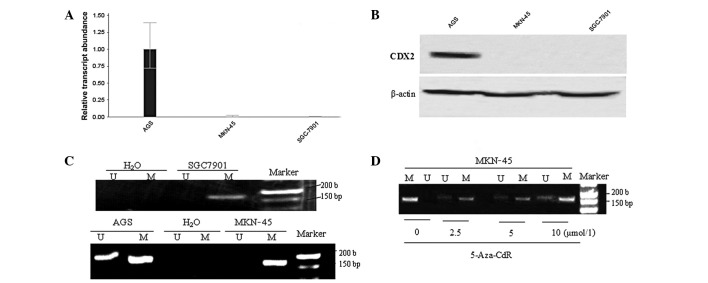
Association between methylation of the CDX2 gene 5′CpG island and gene expression in gastric cancer cells. The expression levels of CDX2 (A) mRNA and (B) protein were markedly high in AGS cells, but absent in MKN-45 and SGC-7901 cells (P<0.01). (C) Methylation analysis of the CDX2 gene 5′CpG island using methylation-specific polymerase chain reaction (MSP) showed hypermethylation in MKN-45 and SGC-7901 cells, however, AGS showed a partial methylation status. (D) Representative MSP analyses of the CDX2 gene in MKN-45 following treatment with different concentrations (0, 2.5, 5 and 10 *μ*mol/l) for 72 h. Water-treated cells served as blank controls. Lane U, amplified product with primers recognizing the unmethylated CDX2 sequence, lane M, amplified product recognizing the methylated CDX2 sequence. CDX2, caudal type homeobox transcription factor 2; 5-aza-CdR, 5-aza-2′-deoxycytidine.

The expression levels of CDX2 mRNA and protein in the human GC cell lines AGS, MKN-45 and SGC-7901 were detected by RFQ-PCR and western blotting. The results suggested that CDX2 mRNA and protein were strongly expressed in the AGS cell line but extremely low or absent in MKN-45 and SGC-7901 cells (P<0.05; Fig. 1A and B).

MSP analysis revealed that the CDX2 promoter region was fully hypermethylated in the GC cell lines MKN-45 and SGC-7901, however, partial methylation status was detected in the GC cell line AGS (Fig. 1C). In the cell line MKN-45, treatment with the demethylating agent 5-aza-CdR for 72 h at different concentrations (0, 2.5, 5 and 10 *μ*mol/l) induced a partial promoter demethylation (Fig. 1D).

### Reactivation of CDX2 and rescue of gene expression by 5-aza-CdR in MKN-45

**Figure 2.**
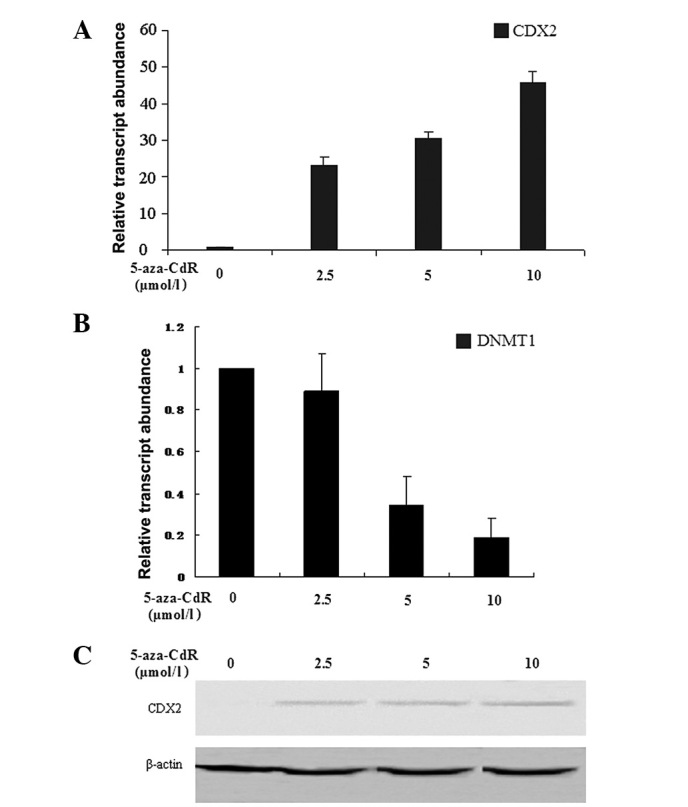
Changes of CDX2 and DNMT1 mRNA expression induced by 5-aza-CdR in MKN-45 cells. (A) Following exposure to 5-aza-CdR for 72 h, a concentration-dependent increase in the level of CDX2 mRNA was observed (P<0.05). (B) A marked concentration-dependent reduction was observed in the levels of DNMT1 mRNA (P<0.05). (C) The expression of CDX2 protein also increased in a concentration-dependent manner (P<0.05). β-actin was used as an internal control. CDX2, caudal type homeobox transcription factor 2; DNMT1, DNA methyltransferase 1; 5-aza-CdR, 5-aza-2′-deoxycytidine..

The human GC cell line MKN-45 was treated with different concentrations (0, 2.5, 5 and 10 *μ*mol/l) of 5-aza-CdR for 72 h. The expression levels of CDX2 and DNMT1 mRNA were detected by RFQ-PCR. The results showed that the expression level of CDX2 mRNA was increased by 5-aza-CdR in a concentration-dependent manner (24.65±2.23, 33.59±1.99 and 48.53±1.77, at 2.5, 5 and 10 *μ*mol/l, respectively) compared with those in the control group (0 *μ*mol/l; P<0.05). A comparable result was observed for CDX2 protein, the expression levels of which also increased (1.42±0.01 and 1.86±0.02, at 5 and 10 *μ*mol/l, respectively) in a concentration-dependent manner compared with those in the other two groups (0 and 2.5 *μ*mol/l; P<0.05). However, the expression level of DNMT1 mRNA showed a marked concentration-dependent decrease (1.00±0.01, 0.89±0.18, 0.34±0.14, 0.19±0.09, at 0, 2.5, 5 and 10 *μ*mol/l, respectively) in MKN-45 cells following exposure to 5-aza-CdR for 72 h (P<0.05; Fig. 2).

### 5-Aza-CdR inhibits MKN-45 cell growth by induction of apoptosis

**Figure 3.**
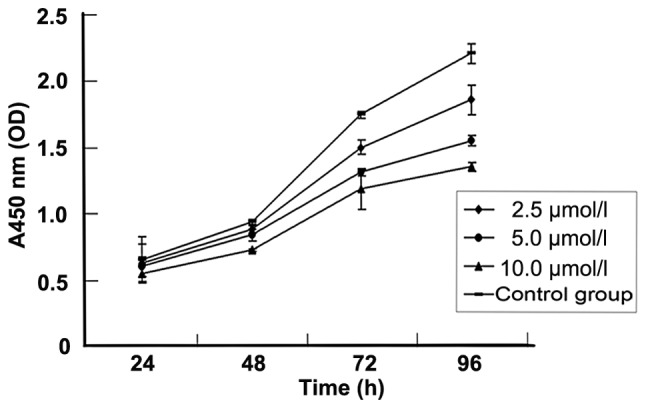
5-Aza-CdR induced cell proliferation inhibition in MKN-45 cells. Cell viability was measured with CCK-8 following the treatment of MKN-45 cells with 5-aza-CdR at different concentrations (0, 2.5, 5 and 10 *μ*mol/l) for 24, 48, 72 and 96 h. The 5-aza-CdR treatment resulted in decreased clonogenic survival, and inhibited cell growth in a concentration-and time-dependent manner (P<0.05). 5-Aza-CdR, 5-aza-2′-deoxycytidine.

To investigate the effect of 5-aza-CdR on the growth of human GC, the MKN-45 cell line was treated with various concentrations of 5-aza-CdR for 96 h and cell viability was detected by the CCK-8 assay. A concentration-and time-dependent growth inhibition of cell proliferation was observed in the MKN-45 cells (Fig. 3, Table III).

**Figure 4.**
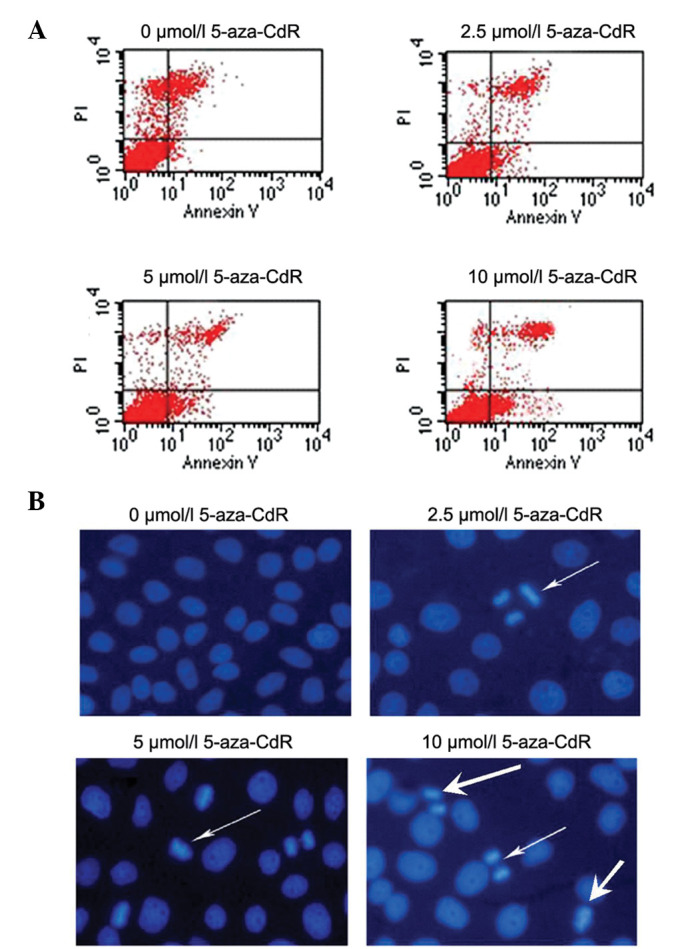
Apoptotic effects of 5-aza-CdR in MKN-45 cells. (A) The percentage of apoptotic cells was scored following cell exposure to 0, 2.5, 5 and 10 *μ*mol/l 5-aza-CdR for 72 h by the Annexin V-FITC/PI assay method, which detects cells in an earlier stage of the apoptotic pathway (the bottom right quadrant) and distinguishes between apoptotic and necrotic cells (the top right quadrant; P<0.05). (B) Cell apoptotic nuclear morphology detected by Hoechst 33258 staining. Following exposure to different concentrations (0, 2.5, 5 and 10 *μ*mol/l) of 5-aza-CdR for 72 h, the MKN-45 cells showed typical apoptotic nuclear morphology. Arrows indicate condensed nuclei. (Original magnification ×200). 5-Aza-CdR, 5-aza-2′-deoxycytidine; PI, propidium iodide.

To examine whether 5-aza-CdR is able to efficiently trigger apoptosis, leading to cytotoxicity against MKN-45 cells, MKN-45 cells were treated with different concentrations of 5-aza-CdR for 72 h. Apoptosis was detected by Annexin V staining FCM assay and Hoechst 33258 staining. As shown in Fig. 4, the data revealed that 5-aza-CdR treatment increased the proportion of apoptotic cells from 0.9±2.3% in pretreated cells to 4.4±2.2, 7.5±1.5 and 15.5±5.0% after 5-aza-CdR treatment at 2.5, 5 and 10 *μ*mol/l, respectively (P<0.05). In addition, Hoechst 33258 apoptosis staining showed morphological changes typical of apoptosis in the nuclear chromatin using fluorescence microscopy. This result strongly suggests that apoptosis rather than necrosis was the mechanism of 5-aza-CdR-induced growth inhibition in the MKN-45 cells.

### 5-Aza-CdR induced apoptosis is mediated via caspase-dependent pathways

In order to examine the role of caspases in the apoptosis induced by 5-aza-CdR, we measured the proteolytic activity of the executioner caspase-3 and the initiator caspase-8 and −9 by measuring Ac-DEVD-pNA, Ac-IETD-pNA and Ac-LEHD-pNA cleavage of MKN-45 cell lysates collected 72 h after 5-aza-CdR treatment. As shown in Table IV, pretreatment with 0, 2.5, 5 and 10 *μ*mol/l 5-aza-CdR caused marked concentration-dependent increases of caspase-3, −8 and −9 proteolytic activities in MKN-45 cells (P<0.05). These results indicate that the activation of caspase-3, −8 and −9 was involved in the 5-aza-CdR-induced cell apoptosis.

## Dicussion

It is generally accepted that the pathogenesis of GC is a multistage process that often takes years, with each stage influenced by environmental, genetic, social and behavioral factors. The characteristics of GC, as Hanahan *et al*(22,23) described, include sustaining proliferative signaling, evading growth suppressors, resisting cell death, enabling replicative immortality, inducing angiogenesis, activating invasion and metastasis, reprogramming energy metabolism and evading immune destruction. Oncogenes and the inactivation of tumor suppressor genes are key molecular factors in the tumor microenvironment in gastric carcinogenesis.

CDX2, as an important nuclear transcription factor, has an essential role in the proliferation and differentiation of intestinal epithelial cells in fetal and adult tissues (24). CDX2 is expressed specifically in colonic and small intestinal mucosa and has been implicated in disorders involving abnormal intestinal differentiation and neoplasia (25,26). The use of CDX2 as an immunohistochemical marker has been described previously in studies of human gastric and colonic cancer (27–30). Several studies have reported that patients with low expression levels of CDX2 in intestinal metaplasia and dysplasia are more likely to progress into GC, and GC patients who were positive for CDX2 expression showed a higher survival rate than those who were CDX2 negative. CDX2 expression levels also gradually during the progression from gastric dysplasia, to early and advanced GCs. In addition, a negative correlation was observed between CDX2 expression and the depth of tumor invasion and lymph node metastasis, suggesting that CDX2 may serve as a powerful predictor for GC (31–37). A recent study showed that the overexpression of CDX2 was capable of inhibiting cell growth and proliferation *in vitro* and effectively inhibited GC progression (38). Therefore, CDX2 acts as a tumor suppressor in the upper gastrointestinal tract. However, the expression of CDX2 gradually declined in the process of intestinal metaplasia-dysplasia-GC (34) and the molecular mechanisms leading to the inactivation of CDX2 remain unclear.

In the present study, our results indicate that the expression levels of CDX2 and DNMT1 mRNA were significantly higher in GC tissues than in non-cancerous tissues. The expression of CDX2 mRNA was correlated significantly with Lauren classification, TNM stage and lymph node metastasis. DNMT1 mRNA expression was significantly correlated with TNM stage, pathological differentiation and lymph node metastasis, which is similar to previous findings reported in the literature (39). Linear correlation analysis showed that the expression of CDX2 mRNA was inversely correlated with that of DNMT1 mRNA in GC. It was then hypothesized that the downregulation of CDX2 in GC was likely to be correlated with the hypermethylation of the CDX2 gene promoter region caused by DNMT1 overexpression.

Recent advances in the field of epigenetics have shown that human cancer cells harbor global epigenetic abnormalities in addition to numerous genetic alterations. Among these epigenetic aberrations, DNA hypermethylation is the one which has been the most extensively studied (40). With regard to our findings, MSP analysis revealed that the CDX2 promoter region was fully hypermethylated in the GC cell lines MKN-45 and SGC-7901, however, partial methylation status was detected in the GC cell line AGS. We conclude that promoter hypermethylation of CDX2 is an epigenetic event in GC that may contribute to epigenetic silencing and result in cancer progression and poor prognosis in patients.

The epigenetic silencing of cancer-related genes has proven to be reversible. Therefore, epigenetic alterations are potential targets of interest for molecular targeted therapy in human malignancies (41). In the current study, we have shown that treatment of the GC cell line MKN-45 with different concentrations of the demethylating agent 5-aza-CdR resulted in partial demethylation of the CDX2 promoter region thus allowing the restoration of a potentially silenced gene expression, while DNMT1 showed a marked concentration-dependent decrease following the exposure of MKN-45 cells to different concentrations of 5-aza-CdR for 72 h. The results of the present study clearly show that the transcriptional inactivation of CDX2 was due to hypermethylation. Notably, MKN-45 cells were treated with 5-aza-CdR at various concentrations and the CCK-8 assay showed that a concentration- and time-dependent growth inhibition of cell proliferation occurred in the MKN-45 cells. Apoptosis analysis revealed that 5-aza-CdR treatment increased the proportion of apoptotoc cells, and morphological changes typical of apoptosis were observed in the nuclear chromatin.

Previous studies have reported (42–45) that the intrinsic mitochondrial or death receptor pathways are able to trigger cell apoptosis, and the mitochondrial apoptotic pathway, including caspase-dependent or independent apoptosis. Caspases are able to cleave essential cellular substrates after aspartic residues and are critical for the initiation and execution phases of apoptosis. Caspase-8 is involved in the death-receptor pathway while caspase-9 mediates the mitochondrial pathway. Once activated, caspase-8 and caspase-9 activate downstream caspase-3, triggering cell apoptosis. Therefore, we measured the activities of caspase-3, caspase-8 and caspase-9 and the results showed that they were all activated, suggesting that the mitochondrial and death-receptor pathways were involved in 5-aza-CdR-induced apoptosis. However, compared with caspase-8, the activity of caspase-9 was higher in response to 5-aza-CdR, suggesting that the apoptosis proceeded mainly via a caspase-dependent intrinsic mitochondrial pathway.

In summary, the results of the current study suggest the detection of CDX2 and DNMTl mRNA will be beneficial in predicting the GC histological type and patient progression, and may also be used as markers in the assessment of the biological behavior of GC. Furthermore, our results confirmed that, in the GC cell line MKN-45, the transcriptional inactivation of CDX2 was due to hypermethylation and the high level of DNMT1. Treatment with a DNMT1 inhibitor rescued the expression of CDX2, inhibited cell proliferation and induced caspase-independent apoptosis. Therefore, our study further emphasizes the importance of the CDX2 gene in gastric carcinogenesis and progression, and a better understanding of DNA methylation is likely to provide us with a potential therapeutic target for GC.

## Figures and Tables

**Table I.** Relative expression of CDX2 and DNMT1 mRNA in gastric cancer and distal non-cancerous gastric tissues (mean ± SD).

Group	CDX2	DNMT1
Non-cancerous gastric tissue	1.34±2.12	5.23±3.66
Gastric cancer tissue	18.43±16.74^a^	40.45±24.45^a^

a P<0.05 vs. distal cancerous gastric tissue. CDX2, caudal type homeobox transcription factor 2; DNMT1, DNA methyltransferase 1.

**Table II.** Correlation between CDX2 and DNMT1 mRNA expression and clinical pathological features of gastric cancer (mean ± SD).

Clinical features	N	CDX2	DNMT1
Age (years)			
<60	25	20.34±3.31	38.25±1.28
≥60	35	16.98±2.21	42.12±3.67
Gender			
Male	42	18.63±2.13	38.57±6.12
Female	18	19.45±4.32	43.10±3.59
Tumor size (cm)			
<5	32	17.35±8.54	41.17±4.38
≥5	28	19.53±5.12	39.65±4.17
Lauren classification			
Intestinal-type	28	29.31±13.15^a^	38.33±2.56
Diffuse-type	32	7.24±1.75	43.43±8.33
TNM staging			
I+II	26	27.50±11.47^a^	29.22±7.21^a^
III+IV	34	10.01±2.39	52.46±8.86
Tumor differentiation			
Good	29	19.37±2.41	30.56±6.04^a^
Poor	31	18.15±2.20	51.53±14.23
Lymph node metastasis			
Absent	19	26.02±8.72^a^	26.37±5.35^a^
Present	41	11.21±2.02	54.37±12.15

a P<0.05 vs. another group with the same clinical features. N, number; CDX2, caudal type homeobox transcription factor 2; DNMT1, DNA methyltransferase 1.

**Table III.** The inhibitory rate (%) for different concentrations of 5-aza-CdR at different time points in MKN-45 cells.

5-Aza-CdR concentration	24 h	48 h	72 h	96 h
0 *μ*mol/l (control group)	-	-	-	-
2.5 *μ*mol/l	5.5±1.5^a^	6.8±2.0^a^	15.1±2.2^a^	17.0±2.2^a^
5.0 *μ*mol/l	9.9±1.8^a^	12.2±2.1^a^	27.0±2.3^a^	31.8±2.4^a^
10 *μ*mol/l	21.2±2.0^a^	25.95±3.1^a^	34.9±5.2^a^	41.3±2.5^a^

a P<0.05 vs. other 5-aza-CdR concentrations at the same time point. Results represent triplicate experiment average ± standard deviation. 5-Aza-CdR, 5-aza-2′-deoxycytidine.

**Table IV.** Change in caspase activity in MKN-45 cells following treatment with 5-aza-CdR for 72 h.

	OD_405_ value		
5-Aza-CdR concentration	Caspase-3	Caspase-8	Caspase-9
0 *μ*mol/l (control group)	0.083±0.003	0.060±0.005	0.069±0.002
2.5 *μ*mol/l	0.087±0.004^a^	0.069±0.001	0.078±0.005^a^
5.0 *μ*mol/l	0.096±0.005^a^	0.075±0.003^a^	0.099±0.001^a^
10 *μ*mol/l	0.101±0.007^a^	0.089±0.004^a^	0.102±0.007^a^

a Significant difference vs. control group. Results represent triplicate experiment mean ± standard deviation. 5-Aza-CdR, 5-aza-2′-deoxycytidine.

## References

[1] Parkin DM, Bray F, Ferlay J, Pisani P (2005). Global cancer statistics, 2002. CA Cancer J Clin.

[2] Leung WK, Wu MS, Kakugawa Y (2008). Screening for gastric cancer in Asia: current evidence and practice. Lancet Oncol.

[3] Bird A (2007). Perceptions of epigenetics. Nature.

[4] Jones PA, Baylin SB (2007). The epigenomics of cancer. Cell.

[5] Turek-Plewa J, Jagodziński PP (2005). The role of mammalian DNA methyltransferases in the regulation of gene expression. Cell Mol Biol Lett.

[6] Mizuno S, Chijiwa T, Okamura T (2001). Expression of DNA methyltransferases DNMT1, 3A, and 3B in normal hematopoiesis and in acute and chronic myelogenous leukemia. Blood.

[7] Lyko F, Brown R (2005). DNA methyltransferase inhibitors and the development of epigenetic cancer therapies. J Natl Cancer Inst.

[8] Silberg DG, Swain GP, Suh ER, Traber PG (2000). Cdx1 and cdx2 expression during intestinal development. Gastroenterology.

[9] Almeida R, Silva E, Santos-Silva F (2003). Expression of intestine-specific transcription factors, CDX1 and CDX2, in intestinal metaplasia and gastric carcinomas. J Pathol.

[10] Kang JM, Lee BH, Kim N (2011). CDX1 and CDX2 expression in intestinal metaplasia, dysplasia and gastric cancer. J Korean Med Sci.

[11] Freund JN, Domon-Dell C, Kedinger M, Duluc I (1998). The Cdx-1 and Cdx-2 homeobox genes in the intestine. Biochem Cell Biol.

[12] Baba Y, Nosho K, Shima K (2009). Relationship of CDX2 loss with molecular features and prognosis in colorectal cancer. Clin Cancer Res.

[13] Mallo GV, Rechreche H, Frigerio JM (1997). Molecular cloning, sequencing and expression of the mRNA encoding human Cdx1 and Cdx2 homeobox. Down-regulation of Cdx1 and Cdx2 mRNA expression during colorectal carcinogenesis. Int J Cancer.

[14] Vider BZ, Zimber A, Hirsch D (1997). Human colorectal carcinogenesis is associated with deregulation of homeobox gene expression. Biochem Biophys Res Commun.

[15] Park do Y, Srivastava A, Kim GH (2010). CDX2 expression in the intestinal-type gastric epithelial neoplasia: frequency and significance. Mod Pathol.

[16] Saad RS, Ghorab Z, Khalifa MA, Xu M (2011). CDX2 as a marker for intestinal differentiation: Its utility and limitations. World J Gastrointest Surg.

[17] Washington K (2010). 7th edition of the AJCC cancer staging manual: stomach. Ann Surg Oncol.

[18] Livak KJ, Schmittgen TD (2001). Analysis of relative gene expression data using real-time quantitative PCR and the 2(-Delta Delta C(T)) method. Methods.

[19] Mao ZB, Zhang JF, Xu Z (2009). Ectopic expression of guanylyl cyclase C in gastric cancer as a potential biomarker and therapeutic target. J Dig Dis.

[20] Herman JG, Graff JR, Myöhänen S (1996). Methylation-specific PCR: a novel PCR assay for methylation status of CpG islands. Proc Natl Acad Sci USA.

[21] Yuasa Y, Nagasaki H, Akiyama Y (2009). DNA methylation status is inversely correlated with green tea intake and physical activity in gastric cancer patients. Int J Cancer.

[22] Hanahan D, Weinberg RA (2000). The hallmarks of cancer. Cell.

[23] Hanahan D, Weinberg RA (2011). Hallmarks of cancer: the next generation. Cell.

[24] Walters JR, Howard A, Rumble HE (1997). Differences in expression of homeobox transcription factors in proximal and distal human small intestine. Gastroenterology.

[25] Tamai Y, Nakajima R, Ishikawa T (1999). Colonic hamartoma development by anomalous duplication in Cdx2 knockout mice. Cancer Res.

[26] Beck F, Chawengsaksophak K, Waring P (1999). Reprogramming of intestinal differentiation and intercalary regeneration in Cdx2 mutant mice. Proc Natl Acad Sci USA.

[27] Barbareschi M, Murer B, Colby TV (2003). CDX-2 homeobox gene expression is a reliable marker of colorectal adenocarcinoma metastases to the lungs. Am J Surg Pathol.

[28] Werling RW, Yaziji H, Bacchi CE (2003). CDX2, a highly sensitive and specific marker of adenocarcinomas of intestinal origin: an immunohistochemical survey of 476 primary and metastatic carcinomas. Am J Surg Pathol.

[29] Moskaluk CA, Zhang H, Powell SM (2003). Cdx2 protein expression in normal and malignant human tissues: an immunohistochemical survey using tissue microarrays. Mod Pathol.

[30] Kaimaktchiev V, Terracciano L, Tornillo L (2004). The homeobox intestinal differentiation factor CDX2 is selectively expressed in gastrointestinal adenocarcinomas. Mod Pathol.

[31] Seno H, Oshima M, Taniguchi MA (2002). CDX2 expression in the stomach with intestinal metaplasia and intestinal-type cancer: Prognostic implications. Int J Oncol.

[32] Mizoshita T, Tsukamoto T, Nakanishi H (2003). Expression of Cdx2 and the phenotype of advanced gastric cancers: relationship with prognosis. J Cancer Res Clin Oncol.

[33] Fan Z, Li J, Dong B, Huang X (2005). Expression of Cdx2 and hepatocyte antigen in gastric carcinoma: correlation with histologic type and implications for prognosis. Clin Cancer Res.

[34] Liu Q, Teh M, Ito K (2007). CDX2 expression is progressively decreased in human gastric intestinal metaplasia, dysplasia and cancer. Mod Pathol.

[35] Song JH, Kim CJ, Cho YG (2008). Genetic alterations of the Cdx2 gene in gastric cancer. APMIS.

[36] Okayama H, Kumamoto K, Saitou K (2009). CD44v6, MMP-7 and nuclear Cdx2 are significant biomarkers for prediction of lymph node metastasis in primary gastric cancer. Oncol Rep.

[37] Qin R, Wang NN, Chu J (2012). Expression and significance of homeodomain protein Cdx2 in gastric carcinoma and precancerous lesions. World J Gastroenterol.

[38] Xie Y, Li L, Wang X (2010). Overexpression of Cdx2 inhibits progression of gastric cancer in vitro. Int J Oncol.

[39] Ding WJ, Fang JY, Chen XY, Peng YS (2008). The expression and clinical significance of DNA methyltransferase proteins in human gastric cancer. Dig Dis Sci.

[40] Sharma S, Kelly TK, Jones PA (2010). Epigenetics in cancer. Carcinogenesis.

[41] Gilbert J, Gore SD, Herman JG, Garducci MA (2004). The clinical application of targeting cancer through histone acetylation and hypomethylation. Clin Cancer Res.

[42] Susin SA, Daugas E, Ravagnan L (2000). Two distinct pathways leading to nuclear apoptosis. J Exp Med.

[43] Wang X, Zhu S, Drozda M (2003). Minocycline inhibits caspase-independent and -dependent mitochondrial cell death pathways in models of Huntington’s disease. Proc Natl Acad Sci USA.

[44] Antonsson B (2004). Mitochondria and the Bcl-2 family proteins in apoptosis signaling pathways. Mol Cell Biochem.

[45] Stefanis L (2005). Caspase-dependent and -independent neuronal death: two distinct pathways to neuronal injury. Neuroscientist.

